# Deceptive Jamming Detection for SAR Based on Cross-Track Interferometry

**DOI:** 10.3390/s18072265

**Published:** 2018-07-13

**Authors:** Qingqing Feng, Huaping Xu, Zhefeng Wu, Wei Liu

**Affiliations:** 1 School of Electronic and Information Engineering, Beihang University, Beijing 100191, China; 18010127731@163.com; 2 CASIC, Hepingli South Street, Dongcheng District, Beijing 100013, China; wuzheming001@126.com; 3 Electronic and Electrical Engineering Department, University of Sheffield, Sheffield S1 3JD, UK; w.liu@sheffield.ac.uk

**Keywords:** deceptive jamming detection, synthetic aperture radar, electronic counter-countermeasure, synthetic aperture radar interferometry

## Abstract

Deceptive jamming against synthetic aperture radar (SAR) can create false targets or deceptive scenes in the image effectively. Based on the difference in interferometric phase between the target and deceptive jamming signals, a novel method for detecting deceptive jamming using cross-track interferometry is proposed, where the echoes with deceptive jamming are received by two SAR antennas simultaneously and the false targets are identified through SAR interferometry. Since the derived false phase is close to a constant in interferogram, it is extracted through phase filtering and frequency detection. Finally, the false targets in the SAR image are obtained according to the detected false part in the interferogram. The effectiveness of the proposed method is validated by simulation results based on the TanDEM-X system.

## 1. Introduction

With its all-day, all weather, long-range, and wide-mapping capabilities, synthetic aperture radar (SAR) has been applied in a wide range of areas [1,2,3,4,5,6]. In practice, the SAR system often suffers from complicated electromagnetic interferences. Meanwhile, a variety of jamming techniques, including barrage jamming and deceptive jamming [7,8,9], were developed to reduce the effectiveness of SAR. Barrage jamming can degrade the quality of SAR image significantly by raising the noise level, while deceptive jamming introduces some false targets to cover useful information or interfere with the target extraction and tracking algorithms employed in SAR [10,11,12].

Generally, deceptive jamming is an effective electronic countermeasure (ECM) technique against the SAR system. The jammer can generate deceptive signals by rapidly estimating the SAR signal parameters, such as carrier frequency, chirp rate, bandwidth, etc. [13,14], or re-transmit the intercepted SAR signals with different time delays [13,15]. In recent years, various deceptive jamming methods were proposed. A large scene deceptive jamming method for space-borne SAR is proposed in [16]. An improved method for SAR scattered wave deception jamming, proposed by Zhao et al. [17], has been successfully applied for enlarging the jamming area. As an extension, the range difference measuring approach has been successfully utilized for deceptive jamming of squint SAR based on multiple receivers [18]. To protect the SAR system from such attacks, several electronic counter-countermeasure (ECCM) strategies were proposed. In [15], radiometric calibration is utilized to identify false signals through establishing a quantitative relationship between target backscattering and SAR image gray values. After changing the relative RCS of known targets, the jammed area is then discovered by analyzing the pixel gray values of the image. However, the speed of SAR imaging is very fast and it is difficult to change the relative RCS of known targets in time. In [19], a detection scheme using dual antennas is proposed, where the false target is detected by cancelling the corresponding pixels in two SAR images with a proper weighting coefficient. However, due to difficulty in deriving the coefficient for each pixel, it is not an easy task to identify the false targets on the scene with hypsography changes [19]. 

The above methods are based on the statistical characteristics of SAR image pixels, especially the amplitude of false signals. However, when the deception reflectors are not strong or the backscattering characteristics of ground targets are complicated, these methods may become less effective. In [20], the influence of SAR deceptive jamming on the InSAR process is studied and it is found that the property of interferometric phase between real targets and false ones are quite different. Therefore, in this paper, to identify deceptive jamming more effectively, a novel approach is proposed based on cross-track interferometry by exploiting the interferometric phase differences between real targets and false ones in the corresponding SAR images.

The proposed method detects deceptive jamming of SAR image with single-pass SAR interferometry. Initially, one antenna serves as a transmitter and two cross-track antennas record the scattered signals simultaneously. Then, the corresponding SAR images are co-registered with each other and the interferogram of the two SAR images are obtained. The real phase, representing terrain features, varies at different positions with the terrain difference, while the false phase approaches a constant value. As a result, the false target can be identified in the interferogram. Finally, the false phase is extracted through frequency detection in the range direction. For more effective extraction, phase filtering is implemented before frequency detection. 

The paper is organized as follows. In Section 2, the principle of deceptive jamming detection and the geometric configuration among two cross-track antennas, jammers and jammed targets are provided. The detection scheme for the proposed method is presented in Section 3 and the basic steps of deceptive jamming extraction are further discussed in Section 4. In Section 5, experimental analysis of the proposed method is provided and conclusions are drawn in Section 6.

## 2. Principle of Deceptive Jamming Detection

In this section, the model for the InSAR deceptive jamming scenario is first introduced, followed by the signal model of deceptive jamming and real signals. Then, the interferometric phase differences between false targets and the surroundings are analyzed. Although, in general, the deceptive scene may be comprised of many false point targets, for simplicity and without loss of generality, an arbitrary single point target is only considered in the following.

### 2.1. Geometric Configuration

**Figure 1:**
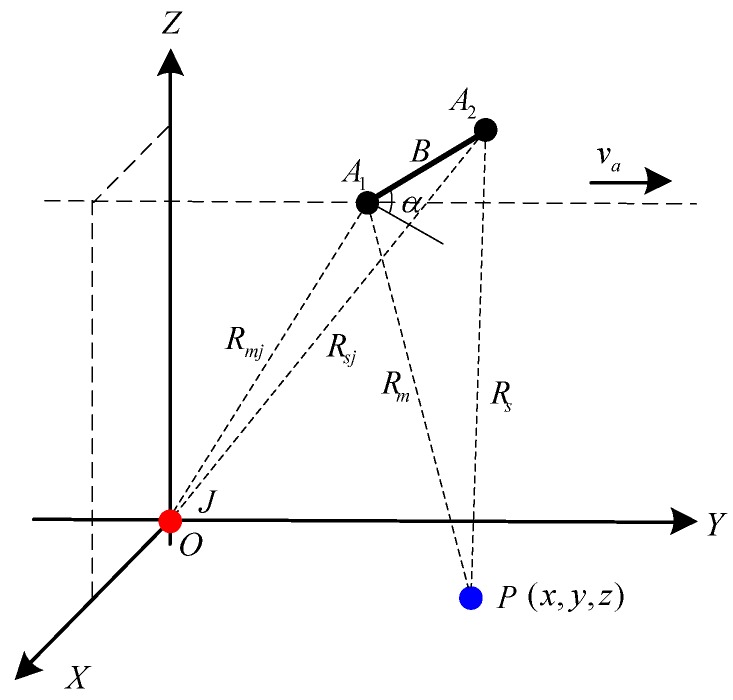
InSAR and jammer geometry.

A schematic illustration for deception detection considered in our work is shown in Figure 1. The jammer J is located at the origin of the Cartesian coordinate system. Points $A_{1}$ and $A_{2}$ represent the two cross-track antennas of the side-looking SAR system. (x,y,z) is the location of an arbitrary jammed target P. Since the SAR platform flies along the *y*-axis at a fixed altitude with a speed of $v_{a}$, we assume that the instantaneous locations of the master antenna $A_{1}$ and the slave antenna $A_{2}$ are $\left(X_{s},v_{a}t_{a},Z_{s}\right)$ and $\left(X_{s}+B\cos(\alpha ),v_{a}t_{a},Z_{s}+B\sin(\alpha )\right)$ at slow time $t_{a}$, respectively. The distances from the master antenna and the slave antenna to the jammer are, respectively, denoted by $R_{mj}(t_{a})$ and $R_{sj}(t_{a})$. $R_{m}(t_{a})$ and $R_{s}(t_{a})$ are the instantaneous slant ranges between the two antennas and the jammed target. α denotes the inclination of the baseline, while B is the length of the baseline. 

Assume the jammer is a stationary point target within one synthetic aperture time, $R_{mj}(t_{a})$ and $R_{sj}(t_{a})$ are calculated by

(1) $$R_{mj}(t_{a})=\sqrt{X_{s}{}^{2}+{\left(v_{a}t_{a}\right)}^{2}+Z_{s}{}^{2}}$$

(2) $$R_{sj}(t_{a})=\sqrt{{\left(X_{s}+B\cos(\alpha )\right)}^{2}+{\left(v_{a}t_{a}\right)}^{2}+{\left(Z_{s}+B\sin(\alpha )\right)}^{2}}$$

Similarly, $R_{m}(t_{a})$ and $R_{s}(t_{a})$, are given by

(3) $$R_{m}(t_{a})=\sqrt{{\left(X_{s}-x\right)}^{2}+{\left(v_{a}t_{a}-y\right)}^{2}+{\left(Z_{s}-z\right)}^{2}}$$

(4) $$R_{s}(t_{a})=\sqrt{{\left(X_{s}+B\cos(\alpha )-x\right)}^{2}+{\left(v_{a}t_{a}-y\right)}^{2}+{\left(Z_{s}+B\sin(\alpha )-z\right)}^{2}}$$

### 2.2. Signal Model

According to [21], the jammer transmits the deceptive jamming signal with a time delay to cover useful information, while the true target reflects echo according to the radar cross section (RCS). As can be seen, the slant range histories for the jamming signal and the real echo are obtained in different ways. After echo analysis and imaging, the final SAR imagery of the deceptive jamming part in the master image and slave image are, respectively, represented by [20]

(5) $$\begin{array}{l} J_{M\_out}(t_{r},t_{a})\\ =GA\sigma (x,y)\sin c\left(t_{r}-\frac{2R_{M}}{c}\right)\sin c(t_{a}-t_{0})\exp\left(-j\frac{4\pi }{\lambda }R_{M}\right) \end{array}$$

(6) $$\begin{array}{rr} J_{S\_out}(t_{r},t_{a})\approx & GA\sigma (x,y)\sin c\left(t_{r}-\frac{R_{M}+R_{S}}{c}\right)\sin c(t_{a}-t_{0})\\ & \cdot \exp\left(-j\frac{4\pi }{\lambda }R_{M}\right)\exp\left(j\frac{2\pi }{\lambda }\left(R_{MJ0}-R_{SJ0}\right)\right) \end{array}$$

with

(7) $$R_{M}=\sqrt{{\left(X_{s}-x\right)}^{2}+{\left(Z_{s}-z\right)}^{2}}$$

(8) $$R_{MJ0}=\sqrt{X_{s}{}^{2}+y^{2}+Z_{s}{}^{2}}$$

(9) $$R_{SJ0}=\sqrt{{\left(X_{s}+B\cos(\alpha )\right)}^{2}+y^{2}+{\left(Z_{s}+B\sin(\alpha )\right)}^{2}}$$

where $t_{r}$ is the fast (range) time, G is the jammer gain factor, A represents the compression gain in 2-D matched filtering, σ(x,y) is the backscattering coefficient of the false point target, $R_{M}$ is the minimum slant range between the master antenna and the target, c is the speed of light, $t_{0}$ is the time corresponding to the nearest distance between the antenna and the point target, λ is the wavelength, and $R_{S}$ is the minimum slant range between the slave antenna and the target. $R_{MJ0}$ denotes the distance between the master antenna and the jammer when the slant range between master antenna and target is $R_{M}$. $R_{SJ0}$ is the distance between the slave antenna and the jammer when the slant range between slave antenna and target is $R_{S}$.

For the imaging output of the real target, the master image and slave image can be expressed as [22]

(10) $$\begin{array}{ll} S_{M\_out}(t_{r},t_{a})= & A\sigma (x,y)\sin c\left(t_{r}-\frac{2R_{M}}{c}\right)\\ & \cdot \sin c(t_{a}-t_{0})\exp\left(-j\frac{4\pi }{\lambda }R_{M}\right) \end{array}$$

(11) $$\begin{array}{ll} S_{S\_out}(t_{r},t_{a})= & A\sigma (x,y)\sin c\left(t_{r}-\frac{R_{M}+R_{S}}{c}\right)\\ & \cdot \sin c(t_{a}-t_{0})\exp\left(-j\frac{2\pi \left(R_{M}+R_{S}\right)}{\lambda }\right) \end{array}$$

### 2.3. Interferometric Phase Analysis

In the interferometric process, an envelope shift between the master image and the slave image is compensated by high precision image co-registration. After co-registration, the interferometric phase of Equations (10) and (11) is derived as [23]

(12) $$\Delta \phi_{real}=-\frac{2\pi }{\lambda }\left(R_{M}-R_{S}\right)$$

Assuming that the slave image in Equation (6) is adjusted in the light of the master image, $J_{S\_out}(t_{r},t_{a})$ after co-registration is rearranged as

(13) $$\begin{array}{ll} J_{S\_out}(t_{r},t_{a})\approx & GA\sigma (x,y)\sin c\left(t_{r}-\frac{2R_{M}}{c}\right)\sin c(t_{a}-t_{0})\\ & \cdot \exp\left(-j\frac{4\pi }{\lambda }R_{M}\right)\exp\left(j\frac{2\pi }{\lambda }\left(R_{MJ0}-R_{SJ0}\right)\right) \end{array}$$

Then, the interferometric phase of Equations (5) and (13) is given by complex conjugate multiplication as [20]

(14) $$\Delta \phi_{deception}=-\frac{2\pi }{\lambda }\left(R_{MJ0}-R_{SJ0}\right)\approx -\frac{2\pi }{\lambda }\left(R_{MJ}-R_{SJ}\right)$$

where $R_{MJ}$ is the minimum slant range between the master antenna and the jammer and $R_{SJ}$ is the minimum slant range between the slave antenna and the jammer. 

Equation (14) indicates that the phase difference of false target depends heavily on the relative position between the jammer and the two antennas, while not related to the terrain of imaging scene. In practice, the jammer position will be unchanged when SAR deceptive jamming occurs, which results in a phase constant for false targets on the interferogram. Namely, once the location of jammer is determined, the phase constant will not vary even if the jammer changes signal type or alters different deceptive regions on SAR image. Furthermore, Equation (12) shows that the surrounding real areas appear as varying InSAR phases due to different surface elevation. Therefore, the false phase is significantly different from the surrounding real area in the interferogram.

**Figure 2:**
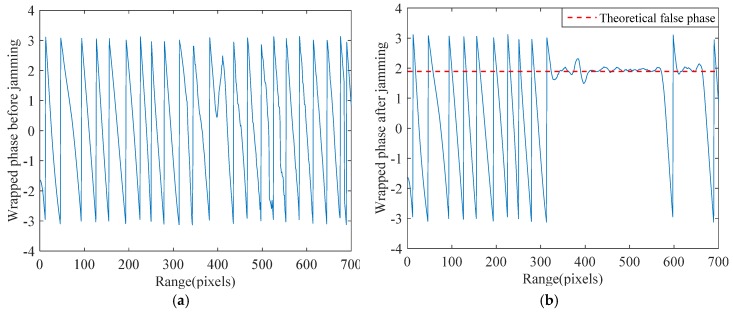
Comparison between the interferometric phase before and after SAR deceptive jamming: (**a**) interferometric phase before jamming; and (**b**) interferometric phase after jamming.

As an example, a series of point targets along the range dimension have been simulated utilizing the system parameters listed in Table 1. After SAR imaging and co-registration of two SAR images, 700 pixels of interferometric phase are generated, as shown in Figure 2a. To jam 331 to 570 pixels and 611 to 660 pixels of the SAR image, we pre-designed the false elevation and repeated the SAR imaging and interferometry process. Then, the interferometric phase after jamming is obtained as shown in Figure 2b. Note that the jammer J is placed on (0,179272.327,0) in the simulation. Additionally, when the minimum distance between the master antenna and the jammer is achieved, the instantaneous locations of the master antenna and the slave one are (0,0,514800) and (0,200,514800), respectively. According to Equation (14), the theoretical false phase can be derived with a value of 1.8891 rad (the red dotted line in Figure 2b). By comparing Figure 2a,b, the false phases significantly differ from the real ones and fluctuate around a constant value as expected, which provides an opportunity to exact the false part from the deceived SAR image.

## 3. Proposed Detection Scheme

With the principle of our proposed method introduced in Section 2, we have the following findings: as the two cross-track antennas are exploited to observe the same area, there exists obvious difference between jammed and non-jammed pixels of interferometric phase; therefore, the cross-track interferometry can be utilized to identify the false targets in the SAR image.

**Figure 3:**
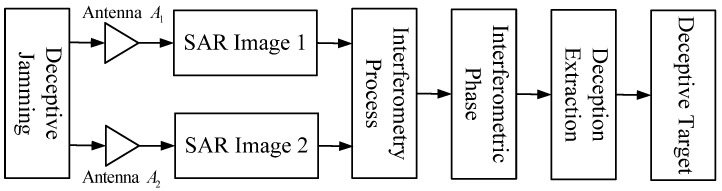
Flowchart of the proposed detection scheme.

Figure 3 shows the flow chart of the proposed detection scheme by two cross-track SAR antennas. After SAR imaging, the dual jammed SAR images are combined to derive the interferometric phase. In the interferogram, the interferometric phase of the false targets can be distinguished from that of the surrounding true elevation. Then, the interferogram is filtered and the false phase can be detected through frequency detection. Ultimately, the corresponding deceptive part in the SAR image is extracted.

## 4. Deceptive Jamming Extraction

**Figure 4:**
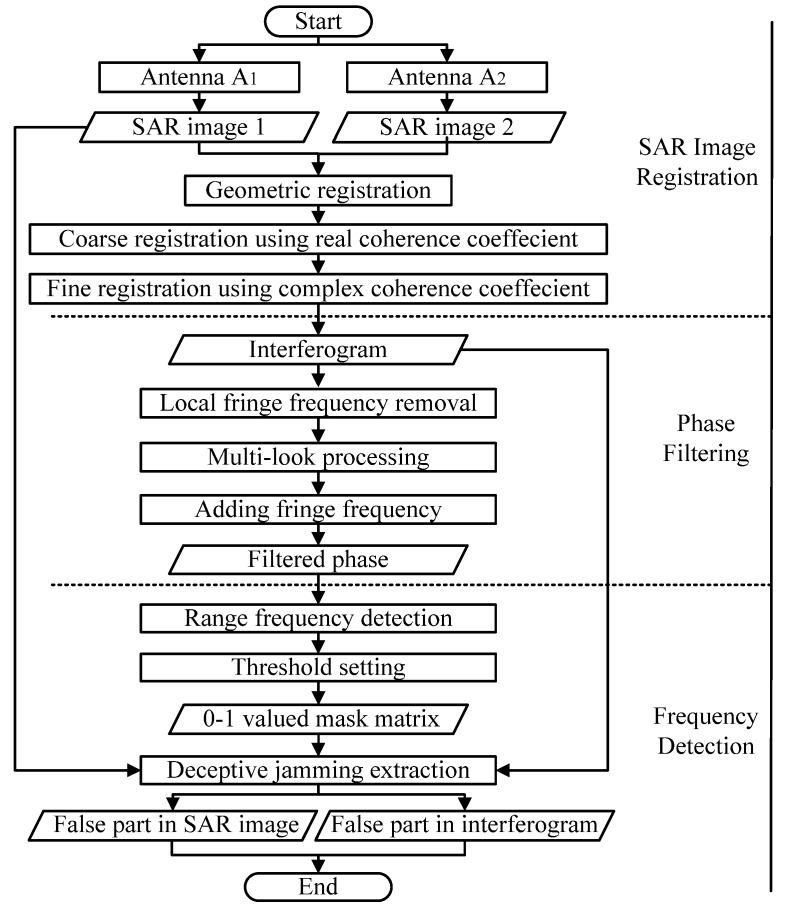
Detailed steps of the proposed SAR deception detection method.

In this part, deceptive jamming extraction based on the interferometric process is further discussed. The basic steps of the proposed method are shown in Figure 4.

To differentiate the deceptive targets from real ones, co-registration of corresponding SAR images is first applied and the interferogram with deceptive jamming is derived. Secondly, phase filtering is carried out to effectively improve the detection accuracy. Then, frequency detection in the range direction is employed to extract the false phase in the interferogram. The false scene in the SAR image is obtained ultimately according to the deduced 0–1 valued mask matrix. 

### 4.1. SAR Image Co-Registration 

Generally, SAR image registration consists of geometric registration and image co-registration. The geometric registration based on imaging geometry and orbit parameters is not necessary unless there is obvious offset or angulation between master and slave image. The co-registration of InSAR images, including coarse co-registration and fine co-registration, is one of the key steps of interferometric processing [24]. Normally, the registration offsets in coarse or fine co-registration depend on the peak position of the correlation function. When calculating offsets, the real and complex correlation functions simplified by Fast Fourier Transform (FFT) can be written as [25]

(15) $$\rho_{r}=norm(\left|FFT^{-1}(FFT(\left|S_{M}(m,n)\right|)\mathrm{FFT}{\left(\left|S_{S}(m,n)\right|\right)}^{*})\right|)$$

(16) $$\rho_{c}=norm(\left|FFT^{-1}{\left(\mathrm{FFT}(\left|S_{M}(m,n)\right|)\mathrm{FFT}(S_{S}(m,n))\right)}^{*}\right|)$$

where norm(⋅) denotes the normalization operator, and $S_{M}(m,n)$ and $S_{S}(m,n)$ represent the master and slave images in the estimated window, respectively.

The research in [26] suggests that the real correlation function is more robust than the complex one in practice, especially in low-coherence regions. Therefore, we choose the real correlation function in coarse co-registration. However, when the two areas have strong coherence and similar scattering properties, the registration error of using real correlation function is approximately $\sqrt{2}$ times that of using the complex correlation function [27]. Meanwhile, after coarse co-registration, the corresponding small grids on the two images are more coherent and have similar scattering properties. As a result, the complex correlation function is selected to fine co-registration.

### 4.2. Phase Filtering

By complex conjugate multiplication of two co-registered images, the InSAR phase with deceptive jamming is obtained. In practice, the quality of interferograms is limited by phase noise induced by co-registration errors, thermal noise, temporal decorrelation, baseline decorrelation and so on [28,29]. To reduce the deception extraction difficulty caused by phase noise, a slope-compensated mean filter [30,31] is applied before frequency detection. 

First, the local fringe frequency estimation is realized by the maximum likelihood (ML) method [32,33]. Subsequently, to preserve the fringes of real targets and strengthen the feature of the false part, the estimated fringe frequency in each filtering window is removed from the original noisy phase. Then, the residual phase part is smoothed based on InSAR multi-look filter processing. Finally, the removed fringe frequencies and the low-noise residual phase are combined to generate the filtered interferogram. The filtering procedure removes most of the phase noise while still preserving the false phase well.

### 4.3. Frequency Detection

After phase filtering, the normal interferometric phase takes the form of continuous fringes with fewer residues, while the false phase in the interferogram appears as a constant in theory. However, in practice, due to the remaining noise and some scattered echo of true targets in the deceptive area, the false phase is not always a constant in the spatial domain, leading to unreliable detection result. Therefore, we apply principle frequency detection to identify the false regions caused by deceptive jamming. 

According to the model of the InSAR phase described in [1], for the normal interferogram, the component of fringe frequency in the radar range direction is related to the flat effect and terrain variety. Therefore, Fourier transform of interferogram in the range direction has continuous changing values in the spectrum. However, the false phase, approximately a constant in the spatial domain, will approach zero in the frequency domain. 

**Figure 5:**
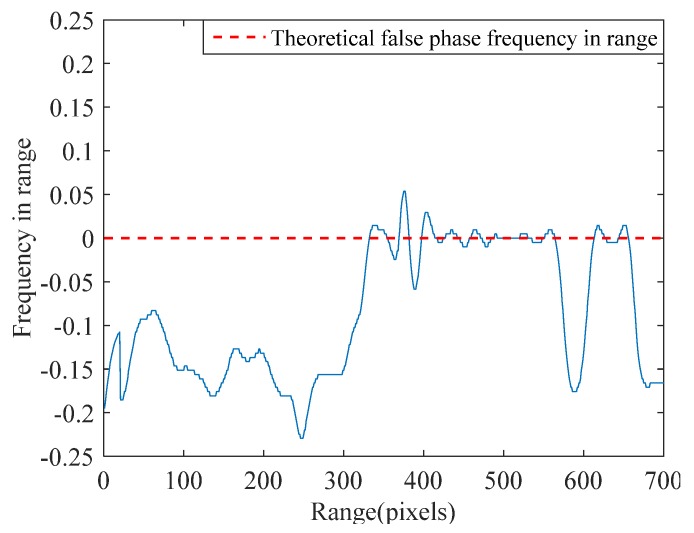
Range frequency of the interferometric phase in Figure 2b.

To derive the range fringe frequency of the detection window, the maximum likelihood (ML) method is employed using Fourier transforms [34]. The range frequency of (2P+1) pixels in the interferogram can be expressed as

(17) $$\hat{f}=\arg\underset{f_{x}}{\max}\left(\left|\sum_{x=a-P}^{x=a+P}\bar{S}\left(x\right)\exp\left(-j2\pi \left(xf_{x}\right)\right)\right|\right)$$

where $\bar{S}\left(x\right)$ represents the filtered complex interferogram, and a denotes the center pixel in the range fringe frequency estimation window. To speed up the optimization of Equation (17), FFT is usually employed. Then, the contours of the deceptive target can be obtained as a constant zero frequency in the range spectrum. For clarity, the range fringe frequency of partially jammed interferometric phase (Figure 2b) is presented in Figure 5. As can be seen, the real phase varies continuously in range spectrum while the false phase from 331 to 570 pixels and 611 to 660 pixels is close to zero, illustrating the validity of the zero-frequency approximation for interferometric phase of false targets. 

Therefore, a 0–1 valued mask matrix can be derived subsequently through threshold setting. Based on the mask matrix, the false area can be extracted from both interferogram and the original SAR image ultimately.

## 5. Experimental Analysis

In this section, to validate the robustness of our method for deceptive jamming detection, simulation results are provided based on the TanDEM-X system [35].

### 5.1. Interferometric Phase Analysis

A terrain with mountainous areas is considered. The system parameters are listed in Table 1. The processing procedures involve SAR imaging, relevant registration, and interferometry. Finally, the interferometric phase with deceptive jamming is derived.

**Figure 6:**
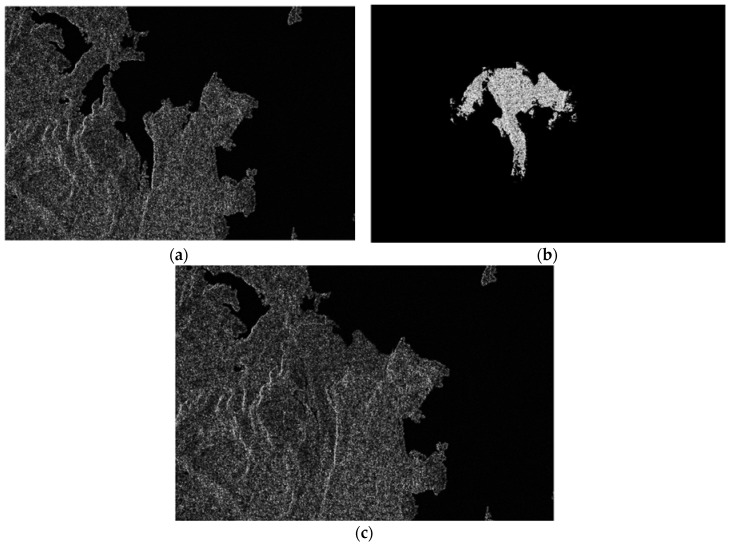
Comparison between the SAR images before and after SAR deceptive jamming: (**a**) master image before jamming; (**b**) deceptive jamming template; and (**c**) master image after jamming.

**Figure 7:**
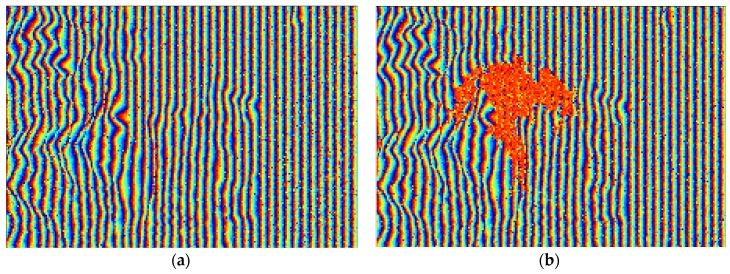
Comparison between the interferograms before and after SAR deceptive jamming: (**a**) interferogram before jamming; and (**b**) interferogram after jamming.

Since the original echo signal and the deceptive jamming signal are received by the two antennas simultaneously, simulation of SAR echo signals with such jamming is first performed. Without loss of generality, only the original master image is shown in Figure 6a. We choose Figure 6b as the deceptive jamming template according to the prior knowledge about targets around the coastal area. Assuming that the jamming to signal ratio (JSR) is 0 dB, the master image with the traditional SAR deceptive jamming transmitted by a single jammer is shown in Figure 6c. Through co-registration and comparing both complex images, the resultant interferometric phase is obtained and depicted in Figure 7b and the interferometric phase without jamming is shown in Figure 7a. By comparing Figure 7a,b, the deceptive part in the interferogram is clearly visible. As can be seen, the false phase of the deceptive target shows as a constant in the interferogram.

### 5.2. Extraction Results

**Figure 8:**
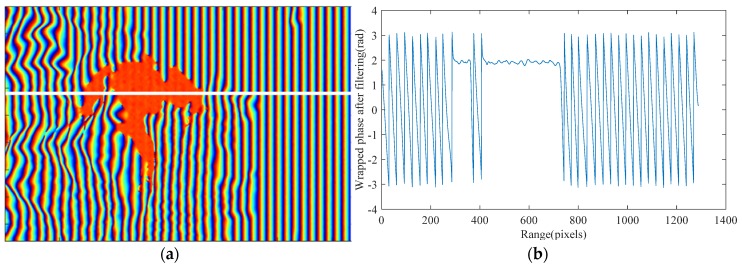
Filtered interferogram with false phase: (**a**) interferogram after filtering; and (**b**) cross-section corresponding to the white line.

A slope-compensated mean filter [30,31] is applied as shown in Figure 8a. Figure 8b shows a cross-section along range direction corresponding to the white line in Figure 8a. It can be seen that the interferometric phase of false targets is close to a constant with only little fluctuations while the surrounding real areas appear as varying phases, which again verifies the previous theoretical analysis.

**Figure 9:**
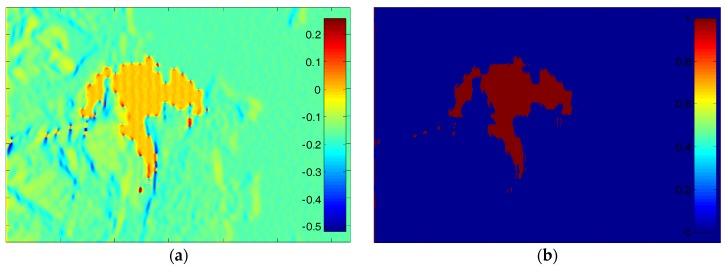
The frequency detection results: (**a**) Fourier transform of interferogram in the range direction; and (**b**) 0–1 valued mask.

Through Fourier transform of interferogram in the range direction, the resultant spectrum is given in Figure 9a. As can be seen, the false phase is close to zero in the frequency domain. Then, setting the threshold as ±0.005 rad, the 0–1 valued mask is obtained as in Figure 9b. Figure 9b also shows the size of deception area detected through our method. 

**Figure 10:**
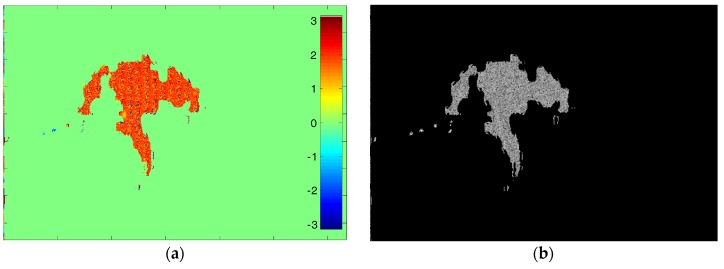
Extraction results: (**a**) false part in the interferogram; and (**b**) false part in the SAR image.

Figure 10 shows the extraction results of the proposed method. Based on the 0–1 valued mask, the false part in interferogram can be detected as in Figure 10a. Correspondingly, the false area in SAR image can also be extracted, as shown in Figure 10b. In summary, the false part in SAR image is detected through interferometry, phase filtering, and frequency detection and result extraction.

**Figure 11:**
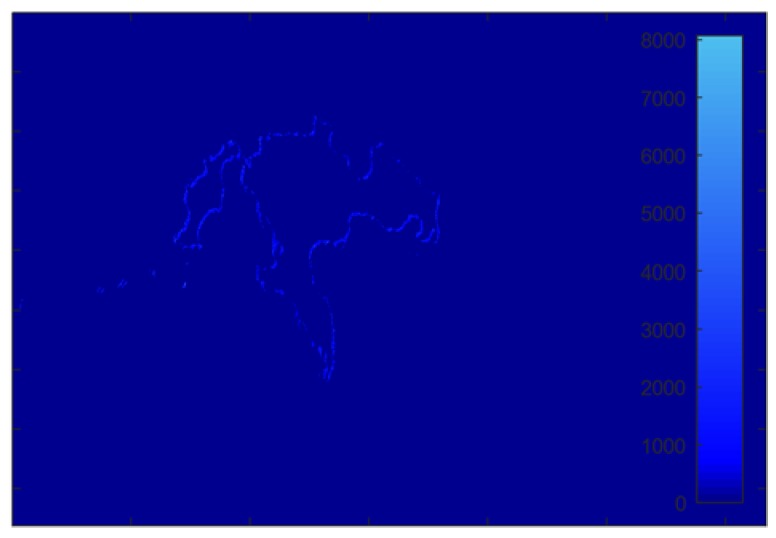
The detection error area in SAR image (absolute value of complex image).

The error detection area of SAR image is provided in Figure 11 and the quantitative results are listed in Table 2. We can see that the correct detection rate of our proposed method has reached 96.84%, while the false alarm rate is only 0.38%, which has again validated the robustness of the proposed method. 

## 6. Conclusions

In this paper, a novel method has been proposed by simultaneously employing two cross-track antennas to detect the deceptive targets in the interferogram, with the following two major findings:

(1) The interferogram produced by combining two SAR images can identify the position of false targets, and the interferometric phases of the false targets related to the jammer position are close to a constant.
(2) Range frequency detection after phase filtering can effectively extract the false phase in the interferogram. With the proposed jamming detection algorithm, the jammed parts in a SAR image can be extracted.

As presented by simulation results based on the TanDEM-X system, the proposed method can detect deceptive jamming effectively and shows great promise in the field of ECCM. Moreover, the detection results also provide a good starting point for possible deceptive jamming suppression.

## Figures and Tables

**Table 1:** Simulation Parameters of the InSAR System.

Parameter	Value
Carrier frequency	9.6 GHz
Chirp rate	$1.3\times 10^{13}$ Hz/s
Pulse duration	10 μs
Sampling frequency	145 MHz
Sensor velocity	7604 m/s
Squint angle	0°
Altitude	514.8 km
Baseline length	200 m
Baseline inclination	0°

**Table 2:** Quantitative Evaluation of our method.

Parameter	Value
Pixel number of SAR image	1,093,016
Pixel number of original false target	58,978
Pixel number of detected false target	61,283
Pixel number of correct detection	57,116
Pixel number of error detection	4167
Correct detection rate	96.84%
False alarm rate	0.38%
